# Body Image Concerns in Patients With Head and Neck Cancer: A Longitudinal Study

**DOI:** 10.3389/fpsyg.2022.816587

**Published:** 2022-03-24

**Authors:** Melissa Henry, Justine G. Albert, Saul Frenkiel, Michael Hier, Anthony Zeitouni, Karen Kost, Alex Mlynarek, Martin Black, Christina MacDonald, Keith Richardson, Marco Mascarella, Gregoire B. Morand, Gabrielle Chartier, Nader Sadeghi, Christopher Lo, Zeev Rosberger

**Affiliations:** ^1^Department of Oncology, McGill University, Montreal, QC, Canada; ^2^Department of Otolaryngology – Head and Neck Surgery, McGill University, Montreal, QC, Canada; ^3^Lady Davis Institute for Medical Research, Jewish General Hospital, Montreal, QC, Canada; ^4^Department of Oncology, Jewish General Hospital, Montreal, QC, Canada; ^5^Department of Otolaryngology – Head and Neck Surgery, Jewish General Hospital, Montreal, QC, Canada; ^6^Department of Otolaryngology – Head and Neck Surgery, McGill University Health Centre, Montreal, QC, Canada; ^7^Department of Nursing, Jewish General Hospital, Montreal, QC, Canada; ^8^Department of Psychology, College of Healthcare Sciences, James Cook University, Singapore, Singapore; ^9^Department of Psychiatry, University of Toronto, Toronto, ON, Canada; ^10^Department of Social and Behavioural Health Sciences, Dalla Lana School of Public Health, Toronto, ON, Canada; ^11^Department of Psychology, McGill University, Montreal, QC, Canada

**Keywords:** body image, cancer, oncology, psycho-oncology, head and neck cancer

## Abstract

**Objective:**

Head and neck cancer (HNC) treatments are known to significantly affect functionality and appearance, leading to an increased risk for body image disturbances. Yet, few longitudinal studies exist to examine body image in these patients. Based on a conceptual model, the current study aimed to determine, in patients newly diagnosed with HNC: (1) the prevalence, level, and course of body image concerns; (2) correlates of upon cancer diagnosis (pre-treatment) body image concerns; (3) predictors of immediate post-treatment body image concerns; and (4) association between body image concerns and levels of anxiety, depression, suicidal ideation, support (i.e., satisfaction with support from physician, social/family wellbeing, and unmet support needs), and alcohol and drug misuse.

**Methods:**

Two hundred and twenty-three (participation rate = 72%), newly diagnosed with a primary HNC were assessed using structured clinical interviews and psychometric measures at three, and 6 months after diagnosis. Primary outcome was 3-month, as it was most salient to body image disturbance. Multiple linear regression analyses were conducted on the potential body image predictors, based on the model.

**Results:**

Sixty-eight percent of patients with HNC (*n* = 148 of 218) presented some level of body image concerns. Body image concerns at baseline (i.e., upon cancer diagnosis, pre-treatment) and post-treatment were significantly related and significantly increased from pre- to post-treatment. Immediately post-treatment (i.e., at 3 month follow-up), 89% (*n* = 132 of 148) presented some level of body image concerns. Correlates of body image concerns in patients with HNC at baseline included: physical symptom burden, difficulties with communication and eating, coping with the cancer diagnosis using denial, suicidal ideation, and having had a past anxiety diagnosis. When controlling for sociodemographic and medical variables, body image concerns in patients with HNC in the immediate post-treatment were predicted by: baseline body image, physical symptom burden, and neuroticism.

**Conclusion:**

This longitudinal study helps identify patients more susceptible to experience body image disturbance following head and neck cancer. Clinicians ought to pay special attention to body image concerns upon cancer diagnosis, physical symptom burden, and neuroticism, and may want to target these factors in future preventive interventions.

## Introduction

Body image is a complex and multifaceted term commonly defined as the subjective perceptions, views, and thoughts of an individual’s own physical appearance (Falk Dahl et al., 2010; Rhoten et al., 2013; Paterson et al., 2016; Shunmuga Sundaram et al., 2019). Body image occurs through both self-observation and noticing how others react to oneself (Falk Dahl et al., 2010; Rhoten et al., 2013; Shunmuga Sundaram et al., 2019). An individual’s attitude toward their body is affected by one’s investment in appearance, meaning the importance of appearance to the individual and self-evaluation relating to the cultural ideals for physical appearance, and the discrepancy they feel between these ideals and their perceived body (Falk Dahl et al., 2010).

Body image disturbance in the cancer context is defined as an individual’s perceived change in appearance and the displeasure and psychological distress felt as a result of this change (White, 2000; Shunmuga Sundaram et al., 2019). As stated by White (2000), “cancer and cancer treatments are, by their very definition, destructive” as the disease and treatments may lead to the loss of body parts, scarring, hair loss, disfigurement, weight changes, and functional limitations that can affect a patient’s self-perceptions and lead to body image disturbance (Vani et al., 2021). The physical changes that occur due to cancer affect patients of all ages and genders and although there are services available to help restore physical appearance, the rates of patients with cancer suffering body image disturbance remains high (between 25 and 77%) (Fingeret et al., 2010; Rhoten et al., 2013; Melissant et al., 2020). Body image concerns have found to be prevalent in many cancer patient demographics particularly in adolescents and young adults diagnosed with cancer (Barakat et al., 2016; Vani et al., 2021). Not only are body image concerns seen in patients of many ages but past research also suggests that patients of a variety of cancers are at risk for developing body image concern including head and neck cancer, colorectal cancer, testicular cancer, gynecological cancer, and breast cancers (Li and Rew, 2010; Sacerdoti et al., 2010; Rossen et al., 2012; Latifi et al., 2020; Song et al., 2021).

Despite the many cancers that have been found to be associated with body image concerns, the majority of research on body image to date in the oncological context has been conducted on patients with breast cancer (Falk Dahl et al., 2010; Paterson et al., 2016; Davis et al., 2020). According to a systematic review conducted by Paterson et al. (2016), in the breast cancer context, cancer patients are highly susceptible to body image concerns due to changes in their physical appearance post-treatment, including the loss or disfigurement of their breasts, scars from surgery, skin changes, hair loss, and chemotherapy induced hormone imbalance leading to weight gain. Overall, body image concerns have been found to be moderated by psychological, social, and environmental factors. In the context of cancer, specifically breast cancer, body image concerns have been found to be impacted by several factors including age, menopausal status, mental health, treatment modality and exercise (Davis et al., 2020). Poorer body image in patients with cancer has been linked to poorer physical and mental health, chronic fatigue, and a reduced quality of life (Falk Dahl et al., 2010; Paterson et al., 2016). Moreover, body image concerns often lead to poor self-esteem, low sexual functioning, depression, and social anxiety and can impact an individual’s quality of life (QoL), personal identity, and self-confidence (White, 2000; Cash, 2012; Rhoten et al., 2013; Juhl et al., 2017; Shunmuga Sundaram et al., 2019). In view of this, it is no surprise that studies have found that individuals with a better conceptualization of their body image tend to cope better with cancer and cancer related treatments as improved mental health and QoL has been consistently found to lead to better treatment and disease outcomes (Carlson and Bultz, 2003; Han et al., 2009; Paterson et al., 2016; Mausbach et al., 2020). Considering the negative health-related outcomes that can occur as a result of the body image concerns held by patients it is important to identify specific moderators or predictors for body image concerns in patients with specific cancers that are most often associated with body image disturbance.

With this in mind, head and neck cancers (HNC) have been found to be particularly associated with body image disturbances (Rhoten et al., 2013). HNC is characterized by malignant tumours originating at the lining surfaces of the oral cavity, sinuses and nasal cavity, pharynx, larynx, and salivary glands (Shunmuga Sundaram et al., 2019). Despite the advances in treatments, HNCs are predominantly diagnosed in advanced stages and often necessitate invasive treatments that involve pain, altered facial appearance (e.g., removal of parts of the face with radial arm free-flap reconstruction, temporary or permanent tracheostomy, visible scars on the face and/or neck), and compromised function in vital and visible areas of eating, speech, and breathing (White, 2000; Rhoten et al., 2013; Nakarada-Kordic et al., 2017; Shunmuga Sundaram et al., 2019; Melissant et al., 2020). Treatment recovery for patients with HNC takes an average of 12 to 36 months and frequently includes long term sequelae (Neilson et al., 2012). These treatment side-effects require major readjustments in physical function, activities of daily living and life roles, particularly affecting one’s identity, social and interpersonal relationships, and often leading to high levels of distress and body image disturbances (Alias and Henry, 2018; Melissant et al., 2020).

Despite the prevalence of body image concerns in patients with HNC, there has been a paucity of studies examining predictors of body image concerns in this population, and a limited number of studies using longitudinal designs (Muzzatti and Annunziata, 2016; Paterson et al., 2016; Vani et al., 2021). In a recent systematic review by Ellis et al. (2019) including only studies examining body image in HNC using psychometric measures, only 2 out of the 17 studies used a longitudinal methodology. The majority of past HNC body image research is cross-sectional, limiting the examination of predictors of body image effects through survivorship, which is detrimental as the few longitudinal studies analyzed by Paterson et al. (2016) found that body image concerns can be present for as long as 12-months post-treatment (Paterson et al., 2016; Vani et al., 2021). The literature suggests that more longitudinal studies are needed to see which characteristics upon diagnosis (baseline) predict body image concerns into survivorship immediately post-treatment or long after (Paterson et al., 2016; Vani et al., 2021).

Furthermore, research in patients with HNC have identified mostly sociodemographic and medical predictors of body image concerns including being female, younger age, a single relationship status, cancer stage, reconstructive surgery, surgical treatments, adjuvant therapies, dysfunction in eating and speech, cognitive difficulties, and lymphedema (Fingeret et al., 2011; Chang et al., 2019; Ellis et al., 2019; Melissant et al., 2020). Additional longitudinal research on body image concerns in patients with HNC using a larger framework of psychosocial variables can help the creation and implementation of effective, timely, and targeted psychosocial interventions that can potentially alleviate body image concerns in patients (Zimmermann et al., 2009; Rhoten et al., 2013; Ellis et al., 2019). In addition to using a longitudinal design as stated above it is essential that research is guided by a framework including a variety of relevant variables. Considering the utility of a HNC specific model for the current study we created a conceptual model specific to HNC.

**FIGURE 1:**
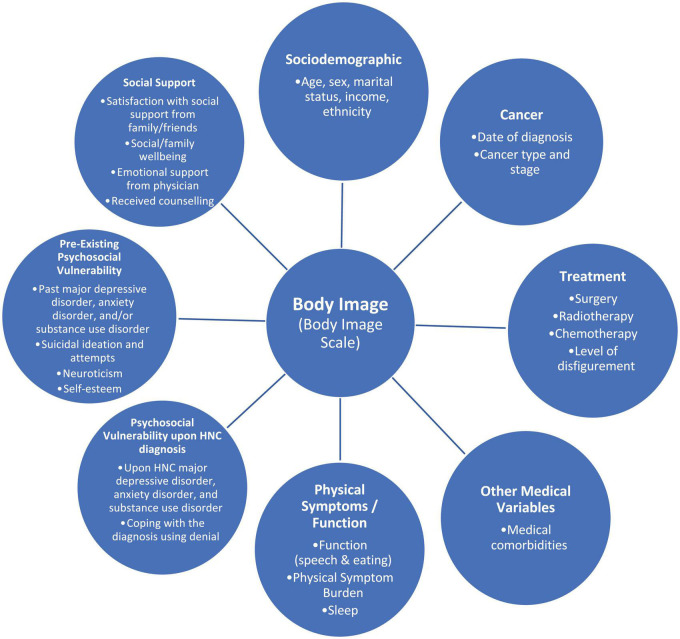
Conceptual model of predictors of body image concerns in patients with HNC.

The conceptual model used in this study is based on the Diathesis-Stress Model (Ingram et al., 1998, 2011) in combination with the Wilson and Cleary Model (Wilson and Cleary, 1995). Wilson and Cleary proposed a conceptual model of health-related quality of life that includes both psychological and biological determinants of outcomes; while the Diathesis-Stress Model posits mental health outcomes as the resultant of an interaction between an individual’s diathesis (i.e., vulnerability) and levels of environmental stress. Our conceptual model includes seven main components: sociodemographic, cancer- and treatment-related variables, other medical variables, physical symptoms/function, pre-existing and upon cancer psychological vulnerability, and social support (see Figure 1). Based on this conceptual model, the current longitudinal study aimed to determine, in patients newly diagnosed with HNC: (1) the prevalence, level, and course of body image concerns from cancer diagnosis to 1-year follow-up; (2) predictors of immediate post-treatment body image concerns (i.e., 3-month post-cancer diagnosis); (3) correlates of baseline (i.e., upon cancer diagnosis, pre-treatment) body image concerns; and (4) association between body image concerns and levels of anxiety, depression, suicidal ideation, support (i.e., satisfaction with support received by physician, social/family wellbeing, and unmet needs for support), and alcohol and drug misuse. We hypothesized that psychosocial vulnerabilities would be early determinants of body image concerns when controlling for sociodemographic and medical variables. We will be analyzing predictors of levels of body image concerns at 3-months post-cancer diagnosis as this is the timeframe when distress is known to be at the highest (Henry et al., 2018a,b).

## Materials and Methods

### Design

This longitudinal prospective study included use of Structured Clinical Interviews for DSM-IV Diagnoses (SCID-I) (First et al., 2002), observer-rated disfigurement measured at baseline (i.e., upon cancer diagnosis, pre-treatment) and post-head and neck cancer treatment, and self-administered questionnaires completed upon cancer diagnosis and at 3-month follow-up. The Structured Clinical Interviews for DSM-IV Diagnoses (SCID-I) were conducted in person by either the research coordinator trained in the approach or the principal investigator (MH; a psychologist with extensive training and experience conducting SCID interviews). The self-administered questionnaires took 60 min to complete at baseline and 45 min at 3 months follow-up. The study received full ethics approval from McGill University Faculty of Medicine’s Institutional Review Board #A05-B24-10B.

### Eligibility and Exclusion Criteria

Eligible patients were adults 18-years and older diagnosed within 2 weeks of a first occurrence of primary head and neck cancer (TNM Classification System), based on tumour board case discussions. Patients diagnosed in the community were re-diagnosed within the recruiting treatment centres and the date of diagnosis was considered from that moment for these patients. All patients were recruited pre-treatment. They needed to be cognitively capable of giving free and informed consent according to physicians, and present a score of 60 or more on the Karnofsky Performance Scale (KPS) upon enrolment and/or an expected survival of at least 6 months according to medical judgement.

### Recruitment

Eligible patients were identified by physicians and nurses of the Department of Otolaryngology – Head and Neck Surgery at McGill University-affiliated hospitals (Jewish General Hospital and McGill University Health Centre – Montreal General Hospital and Royal Victoria Hospital sites). Identified patients were then presented to the research coordinator or contacted by telephone to verify eligibility. The research coordinator arranged for a face-to-face meeting with identified participants in which consent was obtained. A list was kept of patients to follow enrolment and attrition over time. Questionnaires were sent by email and the participants had a 7-day period for completion at each time-point.

### Measures – Outcome

The Body Image Scale (BIS) is the most commonly used measure in body image research (Ellis et al., 2019). It has 10 items measuring general perceptions of bodily appearance in patients with cancer. Respondents indicate their levels of body image concerns on a 4-point Likert type scale: whereby 0 = not at all, 1 = a little, 2 = quite a bit, and 3 = very much (internal consistency 0.93; test-retest reliability 0.70) (Hopwood et al., 2001). A cut-off score of ≥8 on the scale is considered to be clinically significant (Hopwood et al., 2001; Melissant et al., 2018, 2020). Body image scores were characterized as low, low to medium and medium to high based on the mean score of items juxtaposed to the Likert scale anchors (i.e., mild range > 0–0.99, mild to medium 1–1.99, medium to severe 2–3) (Hopwood et al., 2001).

### Predictors

Sociodemographic data were collected through self-administered questionnaires and medical data through chart review. Sociodemographic questions included age, sex, marital status, education, work status, individual and family income, and living alone. Medical variables included cancer stage, cancer type, tumour site, HPV-status, comorbid medical conditions, performance status (ECOG Status) (Oken et al., 1982), and treatments received (surgery, radiation therapy, chemotherapy) with radial forearm free-flap treated as a marker of surgery extent. Other medical variables included use of psychiatric medication and counselling/therapy. Disfigurement was rated using the Observer-Rated Disfigurement Scale for Head and Neck Cancer (Katz et al., 2000).

Physical symptoms were measured by the Functional Assessment of Cancer Therapy – General (FACT-G) (Cella et al., 1993), the Physical Wellbeing subscale (7-items, score range 0–28, internal consistency: >0.70), and item F5 concerning sleep, as well as the FACT-Head and Neck Module (rated on a Likert-scale from 0 to 4 where higher scores represent a better quality of life) (D’Antonio et al., 1996). Function was measured by the Eastern Cooperative Oncology Group (ECOG) scale (Oken et al., 1982), the FACT-HN Module items #7, #11 (swallowing), and #10 (speaking) (D’Antonio et al., 1996).

Pre-existing psychosocial vulnerabilities were examined through the Structured Clinical Interview for DSM-IV diagnoses (First et al., 2002) covering past and upon cancer diagnosis Major Depressive Disorder, Anxiety Disorder, or Substance Use Disorder (inter-rater reliability 0.75 on symptoms and 0.90 on diagnoses). The Eysenck Personality Inventory – Neuroticism Subscale measured neuroticism. The measure includes 12-items with a total score ranging from 0 to 36 and higher scores indicating higher levels of neuroticism (internal consistency: 0.80–0.84) (Barrett et al., 1998). The Rosenberg Self-Esteem Scale measured self-esteem. The measure includes a 10-item scale, with a total score ranging from 0 to 40 and higher scores indicating higher self-esteem (internal consistency: 0.92; test-retest reliability: 0.85–0.88) (Rosenberg, 2016). The Social Support Questionnaire measured social support. The measure includes a 12-item scale, with a total score ranging from 0 to 90 and higher scores indicating higher social support (internal consistency: 0.90–0.93, test-retest reliability: 0.90) (Sarason et al., 1987). Childhood abuse was measured using the Statistics Canadian incidence study of reported child abuse and neglect - 2008 (2010). The Hospital Anxiety and Depression Scale measured anxiety and depression. The scale incudes 14-items with the total score ranging from 0 to 42 and higher scores indicating higher levels of anxiety and depression (internal consistency: 0.78–0.93; test-retest reliability >0.80) (Zigmond and Snaith, 1983). The Beck Scale for Suicidal Ideation measured past and current suicidal ideation. The measure includes 21-items, with the total score ranging from 0 to 42 and higher scores indicating higher levels of suicidal ideation (internal reliability: 0.94) (Beck and Steer, 1991). The Drug Abuse Screening Test and the Rapid Alcohol Problems Screen measured drug and alcohol misuse, respectively. The Drug Abuse Screening Test includes 10 dichotomous items, with the total score ranging from 0 to 10 and higher scores indicating higher level of drug misuse (internal consistency reliability: 0.92) (Skinner, 1982). The Rapid Alcohol Problems Screen includes 5-items with higher scores indicating higher level of alcohol misuse (Cherpitel, 2005). The Brief COPE denial subscale measured coping with a cancer diagnosis using denial and avoidance and includes 2-items ranging from 2 to 8 where higher scores indicate higher denial-based coping (internal consistency: 0.54) (Carver, 1997). Finally, the Supportive Care Needs Survey – Short Form SCNS-SF34 includes 34-items, with the total score ranging from 34 to 170, and higher scores indicating higher levels of unmet needs (internal consistency: 0.86–0.96) (Boyes et al., 2009).

### Statistical Analyses

Analyses were carried out using SPSS Statistics 28. Descriptive statistics were generated for sociodemographic and clinical characteristics, as well as determine prevalence and level of body image concerns at each timepoint. We compared baseline (i.e., upon cancer diagnosis, pre-treatment) and post-levels of body image concerns between time-points (i.e., baseline to 3-months, 3–6 months, and 6–12 months) using paired-sample *t*-tests. Independent sample *t*-tests and correlations were used to measure the association between our identified predictors and outcome (Figure 1). Significant variables were included in a multiple linear regression analysis used to investigate baseline predictors of body image concerns immediately post-treatment. A second multiple regression analysis was used to identify correlates of upon HNC diagnosis levels of body image concerns. Correlations were used to measure association between body image concerns and levels of anxiety, depression, suicidal ideation, support (i.e., satisfaction with support received by physician, social/family wellbeing, and unmet needs for support), and alcohol and drug misuse. For all analyses, a standard alpha level of 0.05 was used.

## Results

Out of the 313 eligible patients with HNC, 223 (71.5%) accepted to participate between September 2012 and September 2015, of which 219 completed the BIS outcome at baseline and 149 at 3-month follow-up see Table 1 for sociodemographic, medical, and clinical characteristics of our sample. Eighty-six percent of participants completed baseline SCID-I interviews and 67.9% at 3 months. Participants who did not complete the follow-up questionnaire at 3 months were not shown to differ on sociodemographic, medical, and psychological variables; except those patients who dropped out presented with significantly lower ECOG functioning (*p* < 0.05).

**TABLE 1:** Sociodemographic, medical, and clinical characteristics of patients newly diagnosed with a first occurrence of head and neck cancer.

Variable	Mean (SD)/ n (%) *n* = 219
Age	63.0 (11.6) range 30–101
Sex: male	151.0 (68.9)
Living alone	71.0 (32.4)
Advanced stage (III/IV)	156.0 (71.2)
Cancer sites	
Oropharynx	80.0 (36.5)
Oral	44.0 (20.1)
Larynx	37.0 (16.9)
Skin	15.0 (6.8)
Nasopharynx	18.0 (8.2)
Unknown primary	12.0 (5.5)
Other (salivary glands, paranasal sinuses, and nasal cavity)	13.0 (5.9)
Cancer type: HPV+	107.0 (54.0)
Treatment: 3 months	
Surgery alone	37.0 (17.2)
Radiotherapy alone	21.0 (9.8)
Chemotherapy alone	5.0 (2.3)
Surgery and radiotherapy	23.0 (10.7)
Radiation and chemotherapy	99.0 (46.0)
Surgery and radiotherapy and chemotherapy	30.0 (14.0)
Major depressive disorder (SCID-I)	
Baseline	13.0 (8.3)
Prior to diagnosis	42.0 (26.9)
Anxiety disorder (SCID-I)	
Baseline	37.0 (23.7)
Prior to diagnosis	47.0 (30.1)
Alcohol use disorder (SCID-I)	
Baseline	8.0 (5.12)
Prior to diagnosis	45.0 (28.8)
Psychological distress (HADS) at baseline	
Anxiety subscale	6.0 (4.6); range 0–20
Depression subscale	3.7 (4.0); range 0–18
Past suicidal ideation	25.0 (11.3)
Neuroticism (Eysenck Personality Inventory) at baseline	7.5 (3.5)
Avoidance coping (Brief COPE) at baseline	
Denial	3.0 (1.4)
Childhood mistreatment < 12 years old	24.0 (13.6)
Parental care in childhood and adolescence (PBI Care)	13.3 (7.9)
Concomitant life stressors < 12 months (SRRS)	3.4 (1.9)
Level of social support (SSQ-6) at baseline	55.3 (26.7)

**FIGURE 2:**
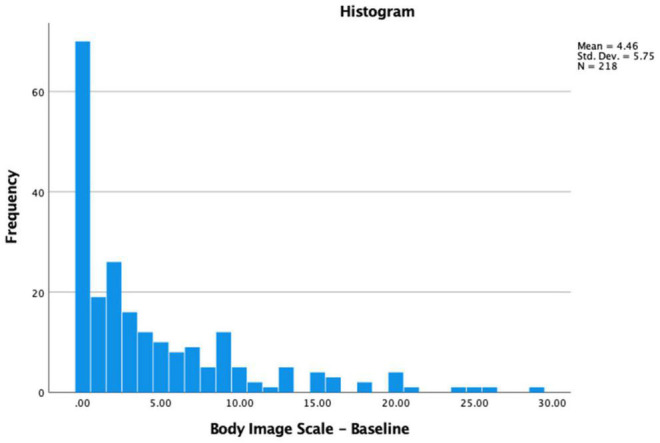
Levels of body image concerns upon HNC diagnosis.

**FIGURE 3:**
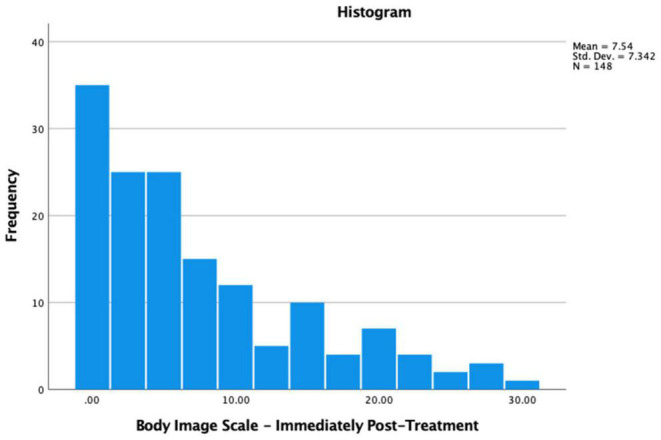
Levels of body image concerns immediately post-treatment in patients with HNC.

Of the participants, 68% (*n* = 148) presented some level of body image concerns at baseline, including 53.7% (*n* = 117) mild, 9.2% (*n* = 20) mild to moderate and 5.0% (*n* = 11) moderate to severe levels. Body image concerns at baseline and post-treatment were significantly related (*r* = 0.55, *p* < 0.001); and significantly increased from baseline to immediately post-treatment (*t*(146) = −7.22, *p* < 0.001; baseline *x* = 4.5, *s.d.* = 5.7; post- *x* = 7.5, *s.d.* = 7.3). Immediately post-treatment, 89% (*n* = 132 of 148) presented some level of body image concerns, including 60.1% (*n* = 89) mild, 20.3% (*n* = 30) mild to moderate and 8.7% (*n* = 13) moderate to severe levels. Body image concerns significantly decreased from immediately post-treatment to 6-month follow-up (*t*(118) = 2.41, *p* = 0.02; immediately *x* = 7.0, *s.d.* = 6.8, 6-month *x* = 5.8, *s.d.* = 6.5), and then stayed the same from 6- to 12-month follow-up (*t*(94) = 0.88, *p* = 0.38; 6-month *x* = 5.6, *s.d.* = 6.4, 12-month *x* = 5.1, *s.d.* = 6.2) (see Figures 2, 3).

When controlling for sociodemographic and medical variables, body image concerns in patients with HNC in the immediate post-treatment (i.e., at 3 months) were predicted by: upon HNC diagnosis body image concerns (*p* < 0.001); physical symptom burden (*p* = 0.003); neuroticism (*p* = 0.01); and difficulties with communication (trend) (*p* = 0.07). These variables accounted 47% of variance in levels of body image concerns (*r* = 0.75; *Adj.r2* = 0.47, *p* < 0.001) (see Table 2).

**TABLE 2:** Predictors of body image concerns immediately post-treatment in patients with HNC.

Measure	B	β	95% CI	*P* value
Body Image Scale – Baseline	0.49	0.36	0.27 – 0.70	<0.001***
FACT physical wellbeing	−0.37	−0.33	−0.61 – −0.13	0.003**
FACT-G sleep	−0.42	−0.07	−1.51 – 0.66	0.44
FACT H&N Module at 3-months	−0.08	−0.09	−0.35 – 0.18	0.55
Patient satisfaction with emotional support from physician	0.11	0.03	−0.39 – 0.60	0.68
FACT functional wellbeing 3 months	0.04	0.04	−0.21 – 0.30	0.74
FACT-G speech 3 months	1.02	0.14	−0.12 – 2.17	0.078^†^
FACT-G eating 3 months	−0.30	−0.06	−1.33 – 0.74	0.57
Coping with denial (COPE denial)	0.36	0.08	−0.39 – 1.11	0.34
Neuroticism	0.33	0.31	0.07 – 0.59	0.012*
Professional counselling 3 months	1.48	0.09	−1.17 – 4.13	0.27
Cancer stage	−0.43	−0.03	−2.35 – 1.48	0.66
Past suicidal ideation	0.91	0.04	−3.09 – 4.92	0.65
HADS anxiety	−0.49	−0.03	−0.43 – 0.33	0.80
HADS depression	−0.06	−0.03	−0.50 – 0.38	0.79
Beck scale for suicidal ideation	−0.04	−0.01	−0.52 – 0.45	0.89
Age (years)	0.03	0.05	−0.09 – 0.15	0.57
Marital status	−0.07	−0.02	−0.68 – 0.53	0.82
Work status	−0.18	−0.07	−0.60 – 0.250	0.41
FACT social/family wellbeing	0.02	0.01	−0.24 – 0.27	0.91

FACT, Functional Assessment of Cancer Therapy; FACT-G, Functional Assessment of Cancer Therapy-General; FACT-HN, Functional Assessment of Cancer Therapy-Head & Neck Module; HADS, Hospital Anxiety and Depression Scale. *p < 0.05, **p < 0.01, ***p < 0.001, ^†^0.10 > p > 0.05.

Correlates of body image concerns in patients with HNC at baseline (i.e., upon cancer diagnosis, pre-treatment) included: physical symptom burden (*p* = 0.002), difficulties with communication (*p* < 0.001) and eating (*p* = 0.05), coping with the cancer diagnosis using denial (*p* = 0.005), suicidal ideation prior to diagnosis (*p* = 0.009), and having had a past anxiety diagnosis (trend) (*p* = 0.058). These variables accounted 47.1% of variance in levels of body image concerns (*r* = 0.74; *Adj.r2* = 0.471, *p* < 0.001) (see Table 3).

**TABLE 3:** Correlates of body image concerns upon HNC diagnosis.

Measure	B	β	95% CI	Value
FACT physical wellbeing	−0.33	−0.29	−0.53 – −0.12	0.002**
FACT sleep	−0.50	−0.10	−1.41 – 0.40	0.28
FACT H&N module	0.09	0.12	−0.09 – 0.26	0.33
Patient satisfaction with emotional support from physician	0.02	0.007	−0.34 – 0.38	0.91
FACT functional wellbeing	0.20	0.22	−0.008 – 0.40	0.060^†^
Difficulties with communication (FACT-G speech)	−2.00	−0.28	−3.08 −−0.91	<0.001***
Difficulties with eating (FACT-G eating)	−1.15	−0.21	−2.17 −−0.12	0.029*
Counselling	−1.43	−0.10	−3.43 – 0.57	0.16
Cancer stage	0.55	0.047	−1.04 – 2.14	0.49
EQPS neuroticism	−0.10	−0.10	−0.28 – 0.09	0.32
FACT family/social wellbeing	−0.07	−0.07	−0.23 – 0.09	0.40
Coping with denial (COPE denial)	0.66	0.19	0.20 – 1.12	0.005**
Past suicidal ideation	−0.46	−0.02	−3.07 – 2.15	0.73
Beck scale for suicidal ideation	0.53	0.19	0.13 – 0.92	0.009**
Past anxiety diagnosis (SCID)	1.85	0.13	−0.06 – 3.77	0.058*
HADS anxiety	0.21	0.16	−0.05 – 0.47	0.11
HADS depression	0.17	0.11	−0.14 – 0.48	0.27
Age (years)	−0.07	−0.12	−0.15 – 0.02	0.13
Sex	1.33	0.11	−0.35 – 3.01	0.12
Marital status	0.03	0.006	−0.46 – 0.51	0.92
Working	0.22	0.11	−0.08 – 0.51	0.15
Social support questionnaire	0.01	0.04	−0.03 – 0.05	0.50

FACT, Functional Assessment of Cancer Therapy; FACT-G, Functional Assessment of Cancer Therapy-General; H&N, Head & Neck; HADS, Hospital Anxiety and Depression Scale; SCID, Structured Clinical Interview for DSM; *p < 0.05, **p < 0.01, ***p < 0.001, ^†^0.10 > p > 0.05.

Body image concerns in the immediate post-treatment were significantly associated with post-treatment levels of depression (*r* = 0.58, *p* < 0.001), anxiety (*r* = 0.51, *p* < 0.001), suicidal ideation (*r* = 0.39, *p* < 0.001), and unmet needs for support (satisfaction with support received by physician, *r* = −0.17, *p* = 0.049, social/family wellbeing, *r* = 0.30, *p* < 0.001 and unmet needs on the SCNS-SF34, *r* = 0.46, *p* < 0.001). All domains of unmet needs on the SCNS-SF34 were affected, with highest association in the psychological and sexuality domains (*r* = 0.53, *p* < 0.001 and *r* = 0.41, *p* < 0.001, respectively), followed by daily living needs (*r* = 0.38, *p* < 0.001), health system and information needs (*r* = 0.27, *p* < 0.001), and patient care and support (*r* = 0.24, *p* = 0.004). Levels of body image concerns immediately post-treatment were not significantly associated with post-treatment alcohol (*p* = 0.43) or drug misuse (*p* = 0.85).

## Discussion

This study contributes to the existing literature in several ways. First, it underlines that concerns around body image are highly prevalent (i.e., in 89% of patients) immediately post-treatment and persistent over time in patients with HNC. Second, most patients present with low levels of body image concerns (60.1%) post-treatment, and clinical levels are present in 29% (20.3% mild to moderate, 8.7% moderate to severe). Third, 68% of patients presented with body image concerns even before treatments commenced, including 53.7% mild, 9.2% mild to moderate and 5% moderate to severe levels.

Body image concerns upon being diagnosed with cancer were associated with physical symptom burden, difficulties with speech/communication and eating, coping with the cancer diagnosis using denial, past suicidal ideation, and having had a past anxiety diagnosis. Immediately post-treatment, levels of body image concerns were predicted by upon HNC diagnosis body image concerns, physical symptom burden, and neuroticism. Furthermore, body image concerns immediately post-treatment were significantly related to post-treatment levels of depression, anxiety, suicidal ideation, and unmet supportive care needs. They were not significantly related to alcohol or drug misuse.

It appears that people diagnosed with HNC may already present with body image concerns even before treatments begin. This may be partly due to physical changes in their bodies, especially physical symptoms and functional changes in speech, communication and eating. This finding is in accordance with past research having found disturbance in speech and eating impairments to be a predictor of body image concern (Fingeret et al., 2011; Chen et al., 2016; Ellis et al., 2019). The mechanisms for this association would need further clarification as it pertains to actual or perceived physical changes, as these changes were measured through patient-reported outcomes in our study. It is unclear if patients more vulnerable to body image concerns would from the start place more emphasis on or be more aware of changes in their bodies. It also becomes clear from this study that one needs to pay attention to patients’ historical background beyond physical changes in appearance, function, and symptom-burden. As such, it appears important to test current models of body image which illustrate interrelated dimensions preceding physical changes such as degree of appearance investment, emotions of self-consciousness or shame and compensatory behaviours (Falk Dahl et al., 2010). Issues around speech would merit further investigation in interfacing with the ability to communicate and social skills, with its implicit working out of issues around stigma and shame (Fingeret et al., 2011; Chen et al., 2016; Ellis et al., 2019). This study also highlights other predisposing historical factors to be investigated in this model such as coping with the cancer diagnosis using denial, past suicidal ideation, and a past anxiety diagnosis.

Neuroticism in particular has been associated with body image concerns in non-HNC populations (Allen and Walter, 2016). Past studies have found that people who exhibit neuroticism (one’s susceptibility to emotional instability) tend to be more self-conscious, care more about how they look, compare themselves to more attractive people, and are often sensitive to rejection causing them to strive for an ideal body (Costa and McCrae, 1992; Davis et al., 2001; Roberts and Good, 2010; Benford and Swami, 2014; Allen and Walter, 2016).

Denial is defined as an unconscious defence against painful aspects of reality in patients with cancer, identified in 4 to 47% and found to be associated with distress and poorer psychological functioning (Vos and de Haes, 2006). Despite research on denial in the cancer context, only one study identified examined denial and its association with body image concerns within the context of patients presenting an eating disorder (Túry et al., 2010). Considering the lack of research on denial and body image and the association found in the current study, future research should examine different coping styles and their association with body image concerns. This is particularly important in patients with head and neck cancer, as use of denial may prevent them from digesting the preparatory information as part of consent for treatments and avoidance may no longer be a helpful coping strategy when reality of treatment impacts hits.

Suicidal ideation and a past anxiety diagnosis as predictors for body image concerns is congruent with past research findings (Fingeret et al., 2011; Rhoten et al., 2013; Paterson et al., 2016; Shunmuga Sundaram et al., 2019). A study examining body image concerns after bariatric surgery, found that patients who scored high on suicidal ideation were more likely to report body image concerns 3 months after bariatric surgery (Pona et al., 2016). Suicidal ideation has been associated with body image concerns in many studies, however, from our review of the literature, suicidal ideation has never been found as a precursor to body image concerns but rather a consequence (Brausch and Muehlenkamp, 2007). Based on our findings, suicidal ideation may be more interconnected to body image concern than previously thought. One may want to investigate how people with past suicidal ideation may present certain features in the face of adversity that would contribute to body image concerns, such as those from theoretical frameworks as the Integrated Motivational–Volitional Model of Suicidal Behaviour (i.e., defeat, humiliation, and entrapment) (O’Connor, 2011), the Attention Mediated Hopelessness (AMH) Theory (i.e., propensity toward depressogenic thinking, ruminative response style, and pessimistic or hopelessness outlook on the future) (Smith et al., 2006), and the Interpersonal Theory of Suicide (i.e., thwarted belongingness and burdensomeness) (Van Orden et al., 2010). The constructs of demoralization and dignity may also merit further attention (Hack et al., 2004; Robinson et al., 2015).

Physical symptom burden was found to predict body image concerns at baseline and immediately post-treatment. In general body image research, having a positive body image often means placing less value on physical appearance and more on physical ability and functionality. This brings up a concern for people who are less physically able due to physical pain or other symptoms associated with a disease as it may change how they experience their bodies (Alleva et al., 2018; Markey et al., 2020). Perceiving something to be “wrong” with one’s body has been associated with poor body image in a study by Markey et al. (2020) which focused on chronic pain. Additionally, Jolly (2011) highlights how symptoms such as pain may leave individuals feeling more uncomfortable and self-conscious based on physical limitations as well as their appearance changes. In addition to pain, fatigue has also been associated with body image concerns in past research, however, very few studies have examined fatigue in the cancer context (Cantarero-Villanueva et al., 2011; Fingeret et al., 2011; Rosenberg et al., 2012). In view of the findings on physical symptom burden and body image, more research is needed to explore the specific symptoms in head and neck cancer that are most associated with body image concerns in order to provide earlier and more effective interventions.

Finally, body image concerns immediately post-treatment were significantly related to levels of depression, anxiety, suicidal ideation, and unmet needs for support in the same time period. They were not significantly related to alcohol or drug misuse. As previously discussed, past research has found depression, anxiety, and suicidal ideation to predict and be associated with body image concerns (Moreira and Canavarro, 2010; Rhoten et al., 2014; Chang et al., 2019; Ellis et al., 2019; Melissant et al., 2020). There may be different pathways to discover in directionality of the relationship between depression, anxiety, and body image, with treatment of depression and anxiety having the potential to alleviate body image concerns and allowing a more nuanced perception of oneself and perhaps of interactions with others.

The findings of this study highlight the importance of using a Diathesis-Stress Model in body image research and a larger set of predictors, including biological, psychological, and environmental factors associated with body image concerns (Wilson and Cleary, 1995; Ingram et al., 1998, 2011).

### Clinical Implications

Patients should be screened upon a HNC diagnosis for baseline body image concerns (BIS), physical symptoms (FACT-G), and neuroticism (Eysenck Personality Inventory – Neuroticism Subscale). Further studies could further explore these dimensions as they relate to body image in order to uncover mechanisms on which to build appropriate interventions.

One can foresee patients identified as having baseline body image concerns be offered an online therapist led intervention covering topics associated with reconnecting to the body, adjusting to post-cancer identity, improving psychosexual functioning, and aspects of cognitive behavioural therapy (Esplen and Trachtenberg, 2020). Patients identified as exhibiting neuroticism may be presented with mindfulness-based cognitive therapy focusing on emotional regulation, flexibility, and the strengthening of social engagements and supports either by family or non-family members (Armstrong and Rimes, 2016; Olawa and Idemudia, 2020). Another intervention can focus on a balance between optimizing physical symptom management (Markey et al., 2020) and function (Chen et al., 2016) before and after treatment, all the while helping patients to change their focus from a preoccupation with their bodies to their adaptation to a new temporary or permanent normal. Patients using denial may be targeted to help improve their capacity to tolerate difficult affect and develop other more adaptive coping such as approach-oriented coping, emotional expression and acceptance (Stanton et al., 2018). Clinicians may want to use the teach-back method (Talevski et al., 2020) with these patients to help better prepare their expectations as to treatment impacts on their body. Improved psychosocial interventions for patients with HNC based on a solid conceptualization of body image disturbance will necessarily improve distress and quality of life (Rhoten et al., 2013).

### Limitations

While this study is the first to investigate body image concerns prospectively in a large cohort of patients with HNC, several limitations are noteworthy. First, patients having dropped-out of the study were more susceptible to have had lower physical function, which may have minimized the prevalence of body image concerns in our sample. Second, while the Body Image Scale was the best measure to use at the time of the study, it was initially developed for patients with breast cancer and includes items that may not be representative of the head and neck cancer experience. In future studies, one may want to use a body image concern measure more specific to HNC such as the recently developed and validated FACT/McGill Body Image Scale – Head & Neck (FACT-MBIS) (Rodriguez et al., 2019a,b). Finally, recruitment in large university settings may limit result generalizability to less resourced contexts.

## Conclusion

Head and neck cancer has significant sequela to functionality and appearance leading to body image disturbance in many. The current study helps clinicians better predict body image disturbance based on upon cancer diagnosis factors such as degree of pre-treatment body image preoccupations, physical symptom burden, and neuroticism. Better understanding how these early determinants interface with later body image concerns would be of merit to tailor interventions addressing this often-neglected component of HNC. Until then, screening for distress, collaborative care models, and patient-health care provider communication skills training (Barth and Lannen, 2011; Alias and Henry, 2018; Deleemans et al., 2020) remain mainstays in HNC oncology clinics.

## Data Availability Statement

The raw data supporting the conclusions of this article will be made available by the authors, without undue reservation.

## Ethics Statement

The studies involving human participants were reviewed and approved by the study received full ethics approval from McGill University Faculty of Medicine’s Institutional Review Board #A05-B24-10B. The patients/participants provided their written informed consent to participate in this study.

## Author Contributions

MH contributed to the conception, design, and analysis of the study as well as supervised the entire project. Along with MH, JA built the model, assisted with study design, and wrote sections of the manuscript. SF, MiH, AZ, KK, AM, MB, CM, KR, MM, GM, GC, NS, CL, and ZR contributed to the conception, design, manuscript revision, and read and approved the submitted version.

## Conflict of Interest

The authors declare that the research was conducted in the absence of any commercial or financial relationships that could be construed as a potential conflict of interest.

## Publisher’s Note

All claims expressed in this article are solely those of the authors and do not necessarily represent those of their affiliated organizations, or those of the publisher, the editors and the reviewers. Any product that may be evaluated in this article, or claim that may be made by its manufacturer, is not guaranteed or endorsed by the publisher.
